# Supplementary material for: Zinc Supplementation Induced Transcriptional Changes in Primary Human Retinal Pigment Epithelium: A Single-Cell RNA Sequencing Study to Understand Age-Related Macular Degeneration
Source: Cells. 2023 Feb 28;12(5):773. doi: 10.3390/cells12050773 (PMC10000409; doi:10.3390/cells12050773)
Supplement: Supplementary file 1 [file cells-12-00773-s001.zip › Supplementary Figure legends.pdf]

Supplementary Figures

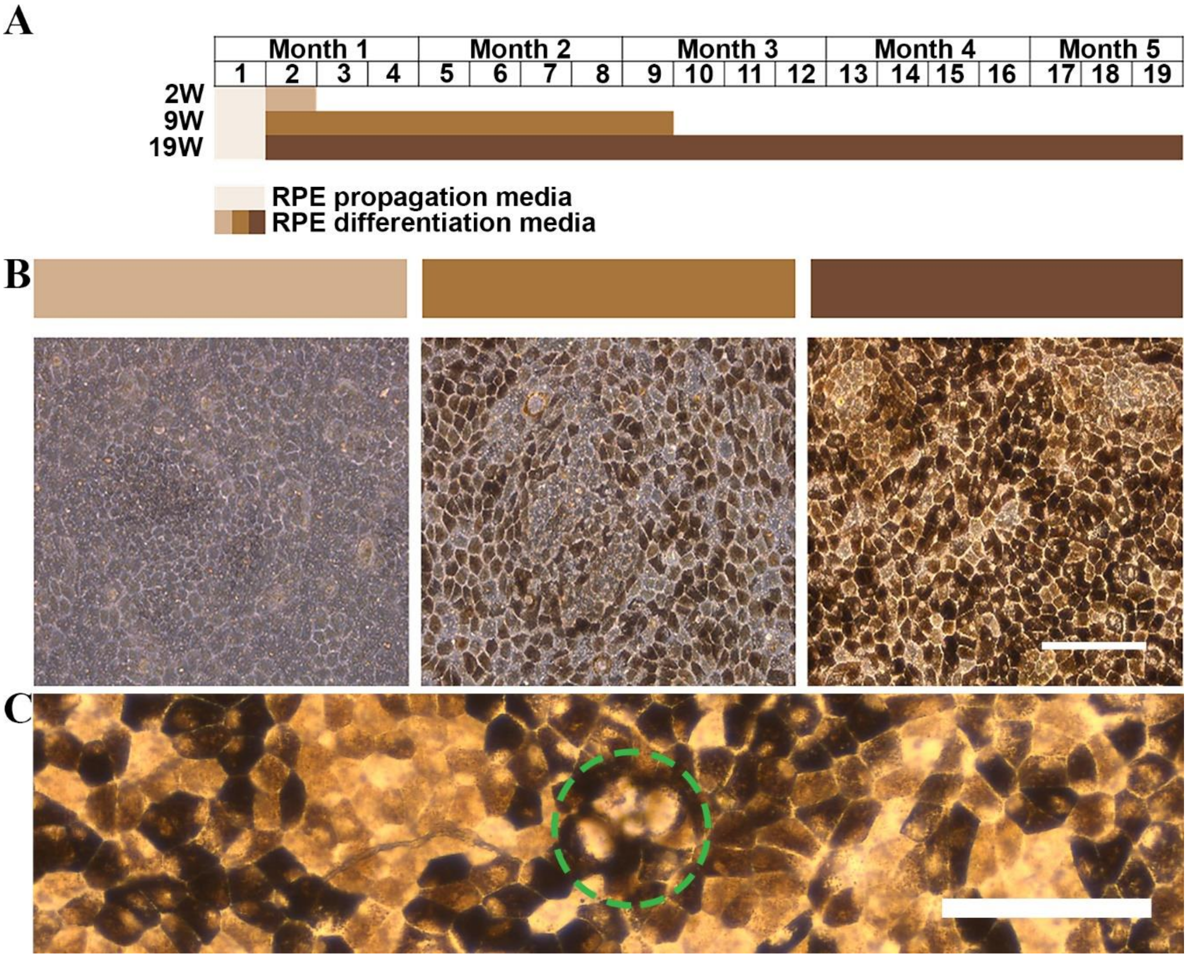

Supplementary Figure S1.: *In vitro* primary fetal RPE culture. Experimental design of follow-up maturing RPE *in vitro* (A), pigmentary changes of *in vitro* RPE over time (B) and representative deposit accumulation in 19 weeks old cells (C).

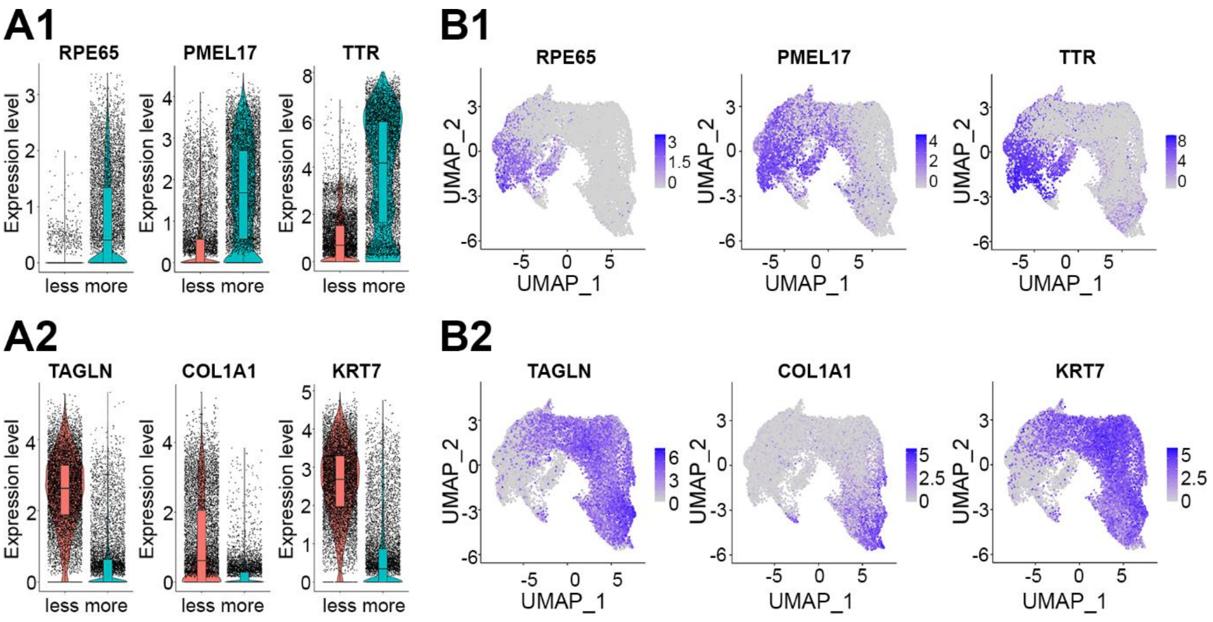

Supplementary Figure S2.: Comparing gene expression patterns of more and less differentiated groups. Three highly expressed marker genes RPE65, PMEL and TTR showed higher expression in the more differentiated cells (A1). Expression of TAGLN, COL1A1and KRT7 were higher in less differentiated cells (A2). The distribution of cells expressing these genes on the UMAP shows the enrichment of cells in the more (B1) or less (B2) differentiated groups. Note: The genes individually did not place every cell into the appropriate groups.

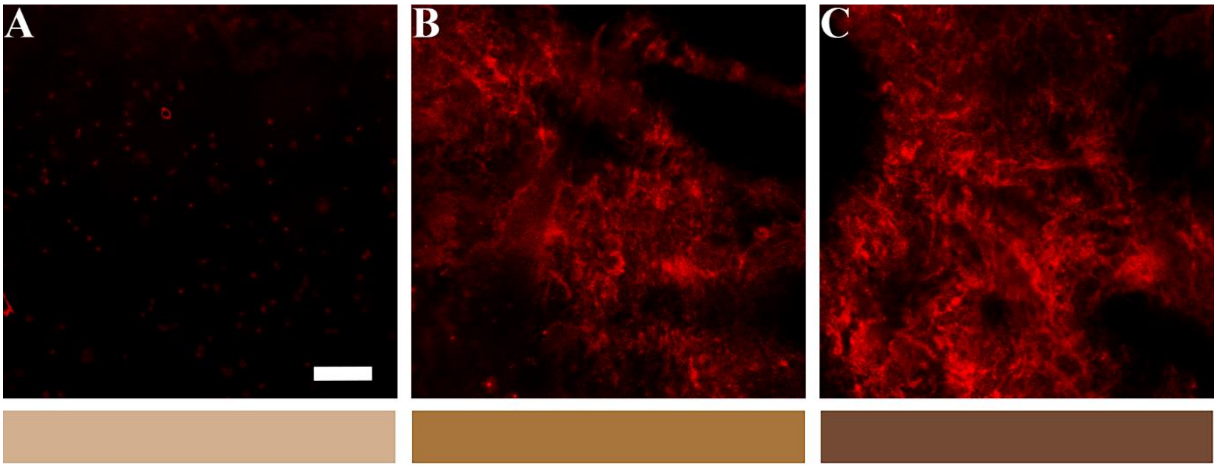

Supplementary Figure S3.: Basal immunolabeling of COL1A1 protein increased in the RPE culture experiments. A, 2 weeks in culture, B, 9 weeks in culture, C, 19 weeks in culture. Scalebar is 10 um.

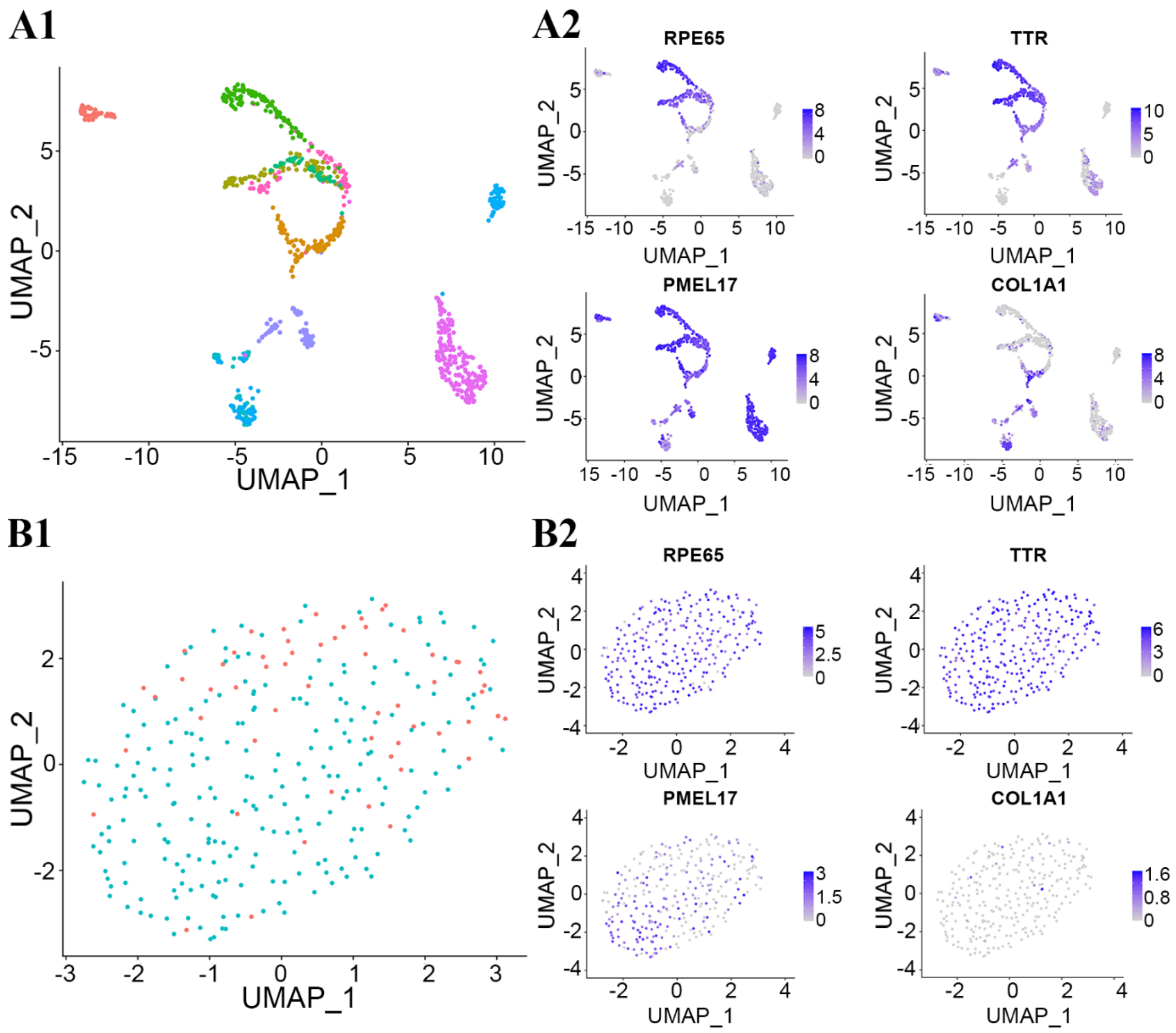

Supplementary Figure S4.: Gene expressional patterns of single-cell populations in the developmental and adult RPE *ex vivo*. Heterogeneity of RPE at single-cell level on *ex vivo* embryonic developmental RPE (A1) and of adult RPE (B1) and gene expression pattern of RPE65, TTR, PMEL and COL1A1 during development (A2) and in adult stage (B2) of RPE.
