# Supplementary figures and images for: Zinc Supplementation Induced Transcriptional Changes in Primary Human Retinal Pigment Epithelium: A Single-Cell RNA Sequencing Study to Understand Age-Related Macular Degeneration
Source: Cells. 2023 Feb 28;12(5):773. doi: 10.3390/cells12050773 (PMC10000409; doi:10.3390/cells12050773)

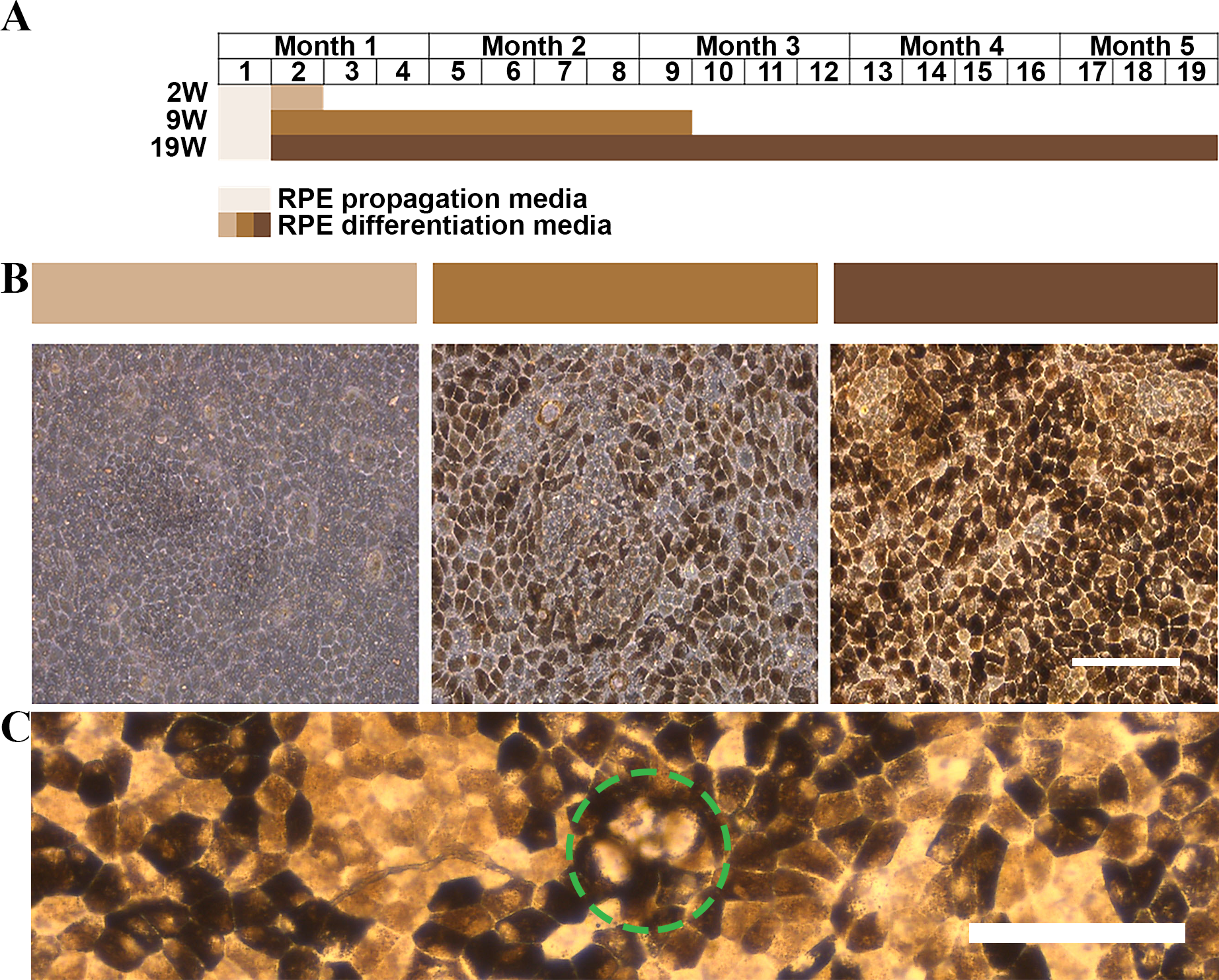

Supplement: Supplementary file 1 [file cells-12-00773-s001.zip › Supplementary Figure 1 BF v3.tif]

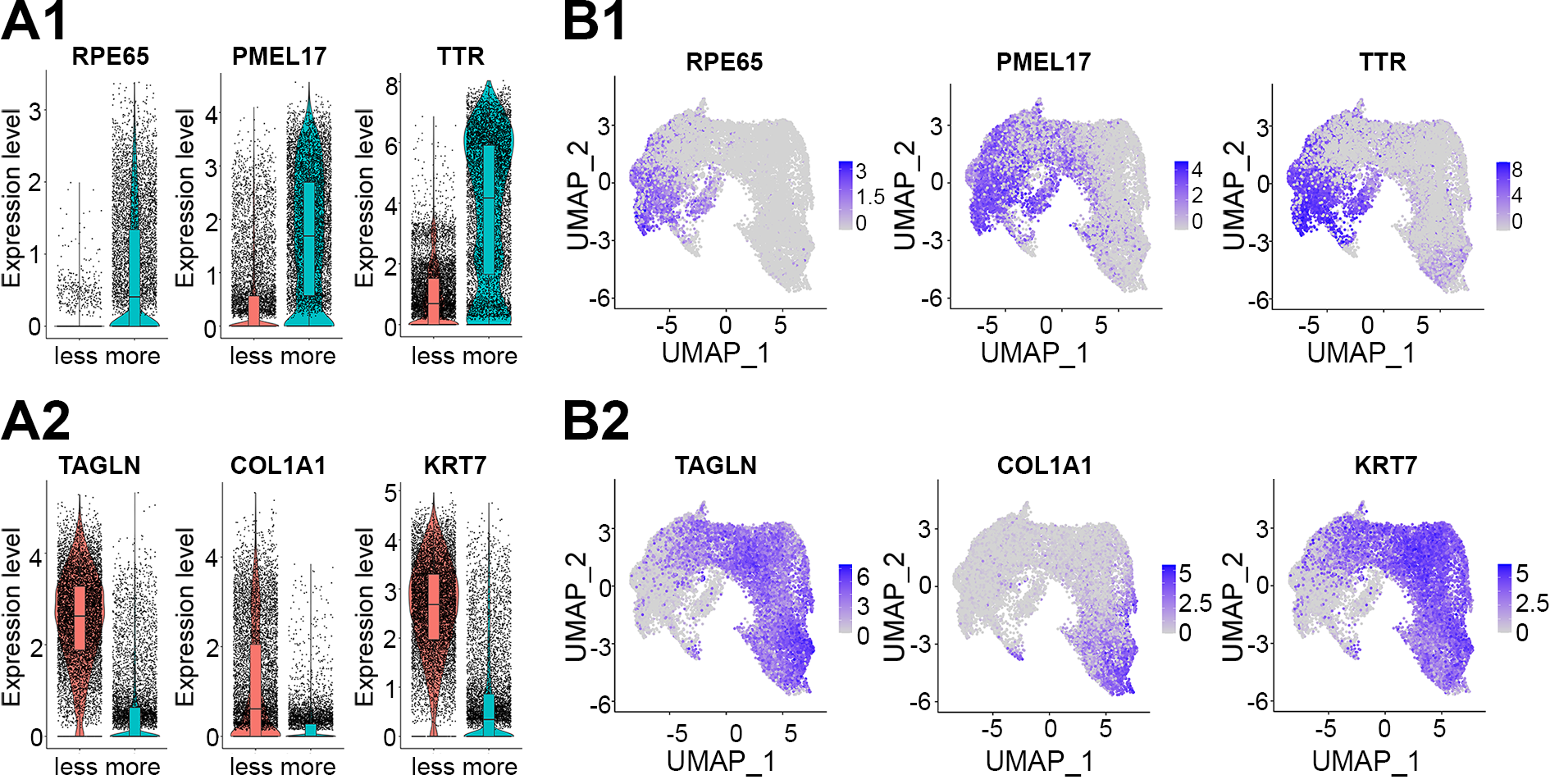

Supplement: Supplementary file 1 [file cells-12-00773-s001.zip › Supplementary Figure 2 TOP3 less and more diff over time.tif]

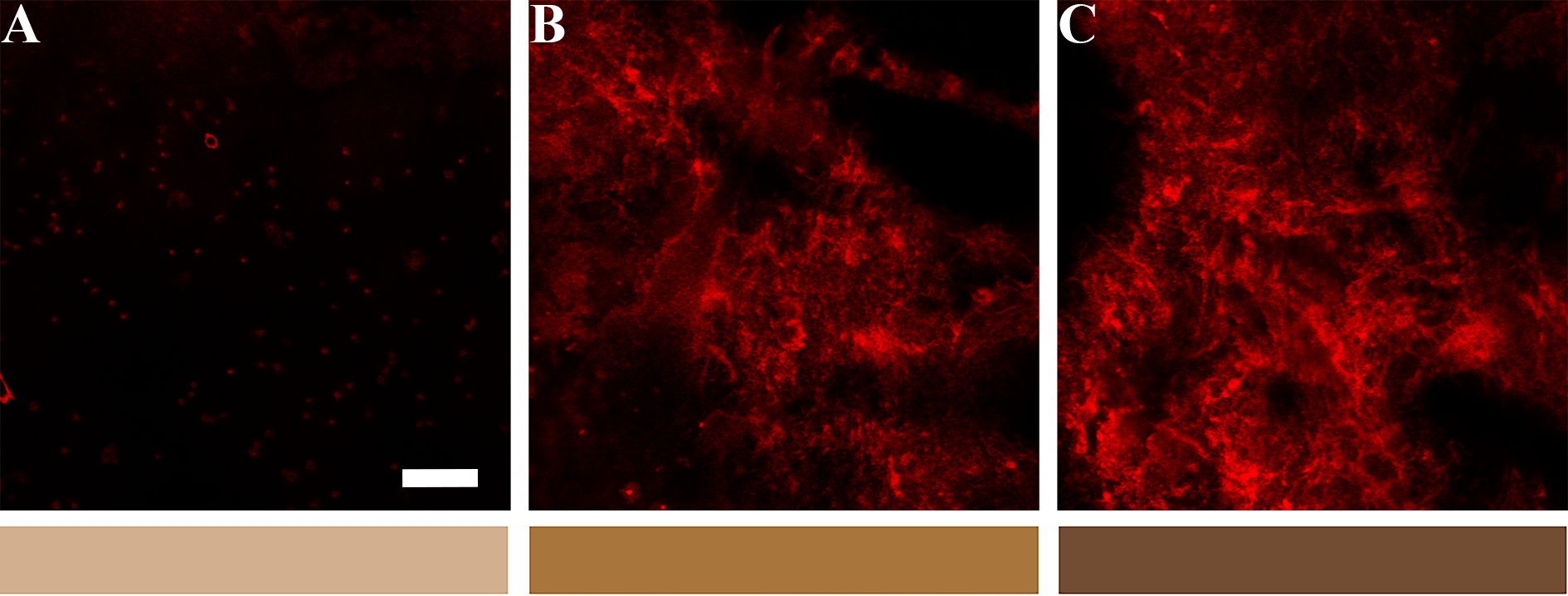

Supplement: Supplementary file 1 [file cells-12-00773-s001.zip › Supplementary Figure 3 COL1A1 labelled.tif]

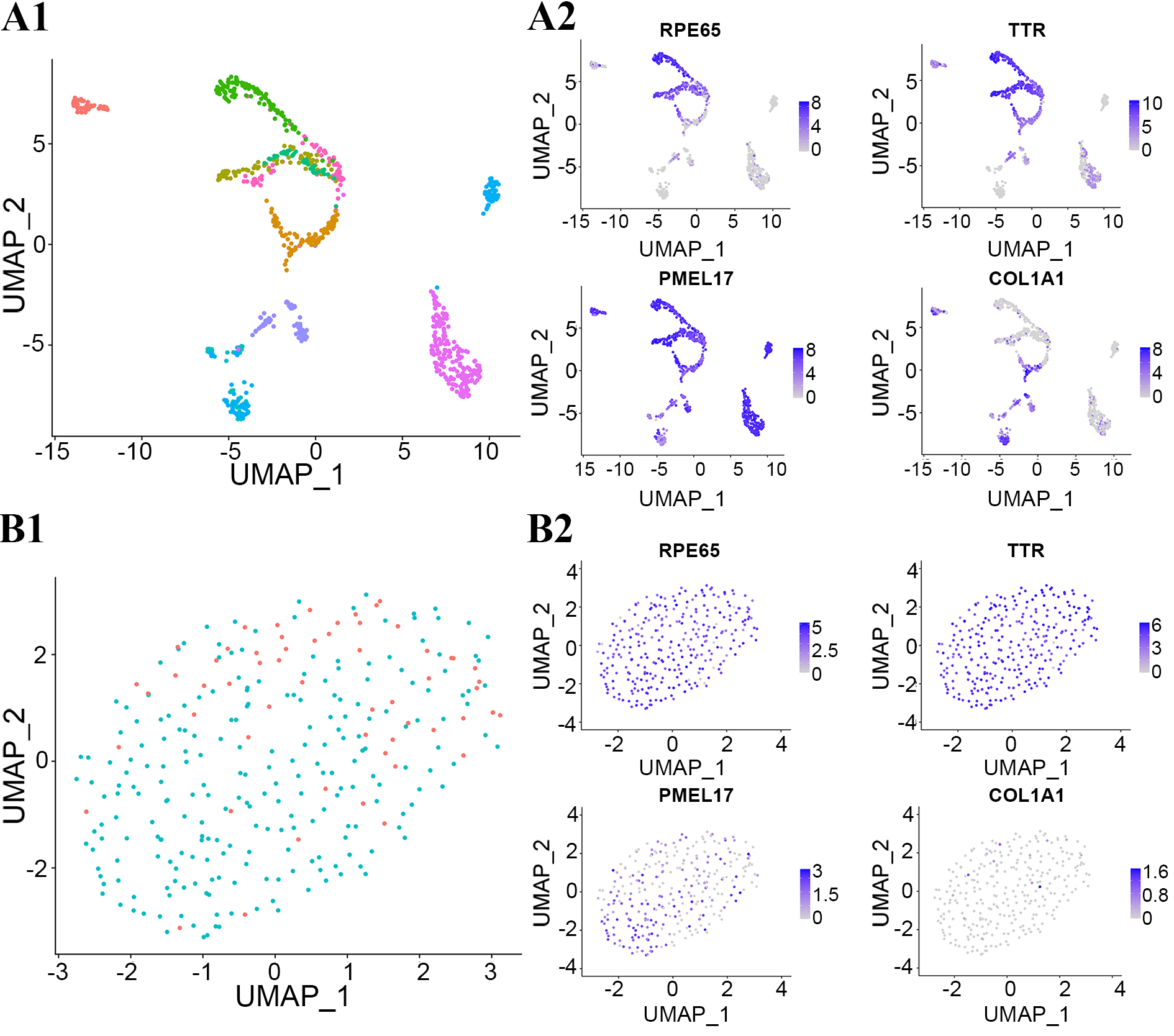

Supplement: Supplementary file 1 [file cells-12-00773-s001.zip › Supplementary Figure 4 in vivo comparison labelled.tif]
